# Pirfenidone and Vitamin D Ameliorate Cardiac Fibrosis Induced by Doxorubicin in Ehrlich Ascites Carcinoma Bearing Mice: Modulation of Monocyte Chemoattractant Protein-1 and Jun N-terminal Kinase-1 Pathways

**DOI:** 10.3390/ph13110348

**Published:** 2020-10-28

**Authors:** Mohamed A. Saleh, Samar A. Antar, Reem M. Hazem, Mona F. El-Azab

**Affiliations:** 1 Department of Clinical Sciences, College of Medicine, University of Sharjah, Sharjah 27272, UAE; 2 Department of Pharmacology and Toxicology, Faculty of Pharmacy, Mansoura University, Mansoura 33516, Egypt; 3 Department of Pharmacology & Toxicology, Faculty of Pharmacy, Horus University, New Damietta 34518, Egypt; santar@horus.edu.eg; 4 Department of Pharmacology & Toxicology, Faculty of Pharmacy, Suez Canal University, Ismailia 41522, Egypt; reem_ahmed@pharm.suez.edu.eg (R.M.H.); mona_elazab@pharm.suez.edu.eg (M.F.E.-A.)

**Keywords:** Fibrosis, Doxorubicin, Pirfenidone, Vitamin D, JNK1, MCP-1, Smad, Ehrlich ascites carcinoma

## Abstract

Treatment of breast cancer with doxorubicin causes numerous side effects, of which cardiac fibrosis is considered the main one. This study was designed to investigate the underlying molecular mechanisms for the potential anti-fibrotic effect of pirfenidone and vitamin D against doxorubicin-induced cardiac fibrosis. Seventy mice carrying solid Ehrlich’s ascites carcinoma (EAC) discs on the ventral side were treated with orally administered pirfenidone (500 mg/kg) and intraperitoneal injection of vitamin D (0.5 µg/kg) either individually or in combination with a doxorubicin (15 mg/kg; i.p.) single dose. All treatments commenced one week post-tumor inoculation and continued for 14 days. Compared to control EAC mice, the doxorubicin group showed a significant increase in heart and left ventricle weights, troponin T, and creatinine kinase serum levels. Furthermore, the doxorubicin group depicts a high expression of monocyte chemoattractant protein (MCP-1), nuclear factor-kappa B (NF-κB), transforming growth factor-beta 1 (TGF-β1), smad3, Jun N-terminal Kinase-1 (JNK1), and alpha-smooth muscle actin (α-SMA). Treatment with pirfenidone or vitamin D significantly decreased all of these parameters. Furthermore, the expression of smad7 was downregulated by doxorubicin and improved by pirfenidone or vitamin D. Furthermore, all treated groups showed a marked decrease in tumor weight and volume. Current data demonstrate that pirfenidone and vitamin D represent an attractive approach to ameliorate the cardiac fibrosis produced by doxorubicin through inhibiting both JNK1 signaling and MCP-1 inflammatory pathways, thus preserving heart function. Further, this combination demonstrated an anti-tumor effect to combat breast cancer.

## 1. Introduction

Breast cancer among women is one of the most common diseases that endanger life [1]. Treatment of breast cancer includes surgery followed by chemotherapy. One of the harmful effects of chemotherapy is fibrosis development. Tissue fibrosis is a complex, multifactorial process caused by healing that follows injury and by pathological changes that disrupt the equation between building-up and the breakdown of extracellular matrix proteins [2]. Treatment of breast cancer with chemotherapeutic agents increases the production of reactive oxygen species (ROS) to a toxic concentration which causes cell damage, inadequate adaptations, and cell death. The heart is more susceptible to ROS injury because the antioxidant resources are lower than other tissues [3].

Doxorubicin is used as one of the routines in curing many malignant tumors [4]. Doxorubicin exerted its cytotoxicity via inhibiting the replication of RNA and DNA [5]. Oxidative stress induced by doxorubicin causes stimulation of myofibroblasts and release of profibrogenic cytokines including transforming growth factor-beta (TGF-β1) and Jun N terminal kinase (JNK1) [6,7]. Furthermore, this condition has a vital role in inducing cardiotoxicity [8].

Pirfenidone is commonly used for the treatment of fibrosis. It exerts both anti-fibrotic and anti-inflammatory activities in a variety of animal and cell-based models [9]. It is an effective agent in the prevention of postoperative fibrosis [10]. Pirfenidone administration reduces the expression of inflammatory markers such as interleukin-1β, “a cytokine that encourages fibroblasts to produce fibrogenic mediators like platelet-derived growth factor (PDGF) and TGF-β1” [11]. However, molecular mechanisms of pirfenidone activity are not fully elucidated, and this manuscript focused on new antifibrotic pathways including JNK1 and MCP-1 pathways.

Vitamin D is an antioxidant that plays a role in attenuation of the myocardial hypertrophy, apoptosis, and inflammation in various experimental models thus preventing the development of heart failure [12]. In addition, vitamin D deficiency may lead to hypertrophy of cardiomyocytes followed by interstitial inflammation and finally fibrosis [13,14]. The anti-inflammatory effect of vitamin D is attributed to brake the nuclear factor kappa B (NF-κB) pathway. Not only acute inflammation, but also chronic inflammation and subsequent fibrogenesis are all regulated by numerous inflammatory molecules such as cytokines, adhesion molecules, and chemokines. The main culprit transcription factor that regulates such molecules is NF-κB [15]. The effect of vitamin D on the JNK1 and MCP-1 signaling pathway may represent a novel mechanism for ameliorating fibrosis.

## 2. Results

### 2.1. Effect on Heart Weight

By weighing the heart, it was found that there was no significant difference between the control EAC and normal group. Furthermore, all treated groups showed a tendency to increase heart weight compared to normal and control EAC but without a significant (*p* < 0.05) difference. Doxorubicin administration resulted in a remarkable (*p* < 0.05) increase in heart weight compared to either control EAC or normal groups. On the other hand, a significant (*p* < 0.05) decrease in weight was observed in all other treated groups except for the combined form of doxorubicin and vitamin D (Table 1).

### 2.2. Effect on Left Ventricle Weight, Tumor Weight, and Tumor Volume

By weighing the left ventricle, it was found that doxorubicin remarkably (*p* < 0.05) increased the weight compared with the control EAC group. On the other hand, there was a significant (*p* < 0.05) decrease in weight in all other treated groups (Table 1). Tumor disks were removed and weighed on day 21. Doxorubicin administration resulted in a significant (*p* < 0.05) decrease in weight compared to the control EAC group. Otherwise, in the vitamin D treated group, the combination forms of doxorubicin with pirfenidone or vitamin D and the co-therapy (doxorubicin, pirfenidone, and vitamin D), showed a significant (*p* < 0.05) decrease in weight compared to the control EAC group. It is worth mentioning that co-therapy treatment could significantly (*p* < 0.05) reduce weight compared to individual treatment with pirfenidone or vitamin D (Table 1). Tumor volume was determined based on caliper measurements. The greatest value of tumor volume was observed in the control EAC group. All treated groups showed a significant (*p* < 0.05) decrease in tumor volume compared to the control EAC group. Co-therapy (doxorubicin, pirfenidone, and vitamin D) revealed a further significant (*p* < 0.05) decrease in tumor volume in comparison with individual pirfenidone or vitamin D treatment (Table 1).

### 2.3. Effect on Serum Level of Cardiac Troponin, Creatine Kinase (CK), and Creatine Kinase Myocardial Band (CK-MB)

**Figure 1:**
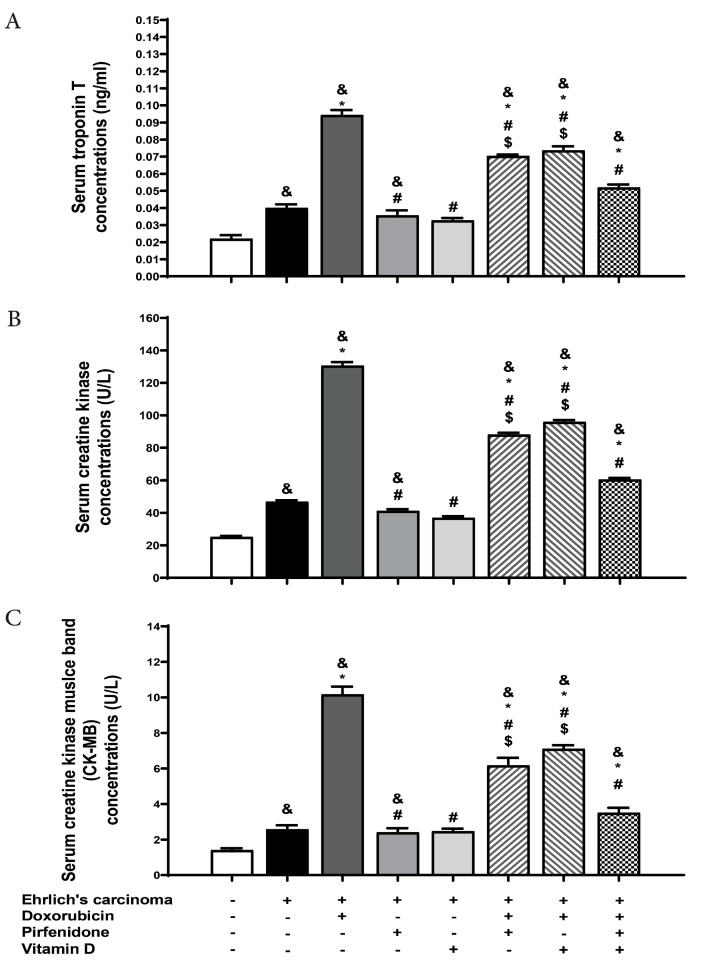
Effect of individual treatment with doxorubicin (15 mg/kg; i.p.), pirfenidone (500 mg/kg; p.o.) or vitamin D (0.5 µg/kg; i.p.) and their combinations on the levels of biochemical markers (**A**) serum troponin and (**B**) creatine kinase (CK) (**C**) CK-MB in EAC bearing mice on day 21. Results are expressed as mean ± SEM. All data were analyzed using ANOVA followed by Bonferroni’s post-hoc test at *p* < 0.05. ^&^ *p* < 0.05 with respect to normal, * *p* < 0.05 with respect to control EAC, ^#^ *p* < 0.05 with respect to doxorubicin. ^$^ *p* < 0.05 with respect to the co-therapy of doxorubicin, pirfenidone, and vitamin D (*n* = 5).

Cardiac troponin release is predictive of prognosis in myocardial infarction and other forms of heart disease. Also, creatine kinase is a very specific and highly sensitive marker for myocardial damage and is commonly used in clinical practice. The obtained results revealed a significant (*p* < 0.05) rise in troponin levels in the control EAC group compared to the normal group. Also, the control EAC group manifested a significantly (*p* < 0.05) high level of CK compared to the normal group. Administration of doxorubicin significantly (*p* < 0.05) increased troponin and CK levels compared to the control EAC or normal groups. However, all treated groups resulted in a significant (*p* < 0.05) decrease in serum troponin compared to doxorubicin. Moreover, a significant (*p* < 0.05) increase in troponin level was observed with all treated groups compared to the normal group. The combination of doxorubicin with either vitamin D or pirfenidone augmented the level of troponin with a significant (*p* < 0.05) difference compared to control EAC. According to the CK level, there was a significant (*p* < 0.05) decrease following administration of pirfenidone or vitamin D daily either individually or in combination forms with doxorubicin compared to doxorubicin. Interestingly, troponin level was significantly diminished after the co-therapy of doxorubicin, pirfenidone, and vitamin D treated group compared to the combination forms of doxorubicin with pirfenidone or vitamin D. CK levels were diminished after the co-therapy of doxorubicin, pirfenidone, and vitamin D compared to the combination forms of doxorubicin with vitamin D (Figure 1). Individual treatment with pirfenidone or vitamin D showed no changes in CK-MB levels compared to normal and control EAC groups. The doxorubicin group presented a significant increase in (*p* < 0.05) CK-MB level which was reduced significantly after combination with pirfenidone or vitamin D. The combined doxorubicin, pirfenidone, and vitamin D-treated group exhibited a significant reduction in serum CK-MB levels when compared to the treatment group of either doxorubicin + pirfenidone or doxorubicin + vitamin D. (Figure 1).

### 2.4. Effect on the Expression of NF-κB and Monocyte Chemoattractant Protein (MCP-1) in Heart Tissue

**Figure 2:**
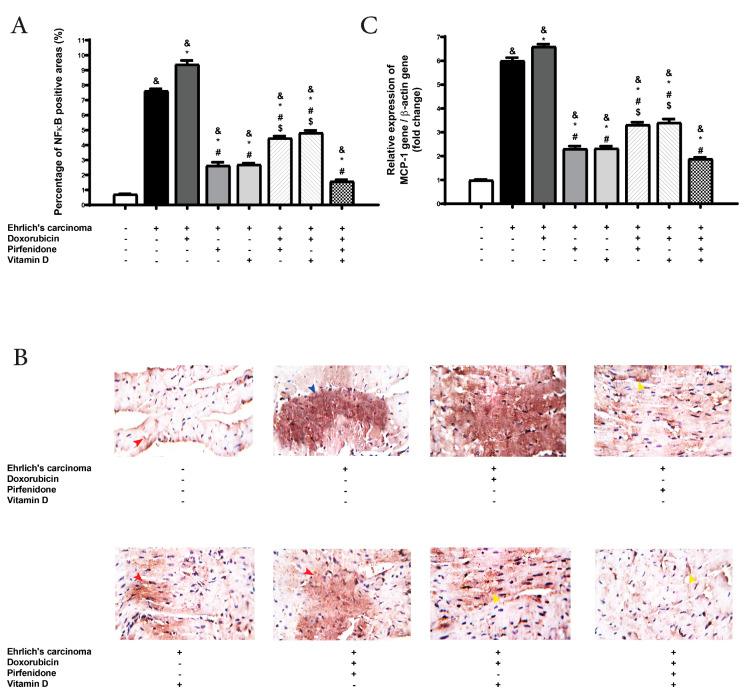
Effect of individual treatment with doxorubicin (15 mg/kg; i.p.), pirfenidone (500 mg/kg; p.o.) or vitamin D (0.5 µg/kg; i.p.) and their combinations on cardiac expression of NF-κB, and MCP-1 in EAC bearing mice on day 21. The bar chart demonstrates NF-κB. (**A**) A graph showing the density of immunostaining for NF-κB in experimental groups. (**B**) Photomicrographs showing cardiac expression of NF-κB. (**C**) RT-PCR measurement of MCP-1 in cardiac tissue. ^&^ *p* < 0.05 with respect to normal, * *p* < 0.05 with respect to control EAC, ^#^ *p* < 0.05 with respect to doxorubicin. ^$^ *p* < 0.05 with respect to the co-therapy of doxorubicin, pirfenidone, and vitamin D (*n* = 5).

The expression of NF-κB stained cells was evaluated in tissues as a marker of inflammation. Also, MCP-1 induced expression of other inflammatory cytokines, recruitment of macrophage, and inflammatory cells into cardiac tissue. The expression of NF-κB was raised in control EAC significantly (*p* < 0.05) compared to normal. A significant (*p* < 0.05) magnification in NF-κB expression was detected in all treated groups compared to the normal group. Also, the control EAC group and all treated groups revealed a remarkable (*p* < 0.05) upregulation in MCP-1 gene expression in heart tissue compared to the normal group. The doxorubicin group showed significant (*p* < 0.05) upregulation in NF-κB and MCP-1 expressions compared to control EAC. On the other hand, there was a significant (*p* < 0.05) downregulation in MCP-1 and NF-κB expressions following treatment to pirfenidone or vitamin D, either individually or in combination with doxorubicin when compared with doxorubicin and control EAC group. At the same time, the co-therapy (doxorubicin, pirfenidone, and vitamin D) showed a remarkable (*p* < 0.05) downregulation in MCP-1 and NF-κB expressions compared to the doxorubicin group, control EAC, and the combination forms of doxorubicin with pirfenidone or vitamin D (Figure 2).

### 2.5. Effect on Transforming Growth Factor Beta 1 (TGF-β1), smad3, smad7, and JNK1 Expression

**Figure 3:**
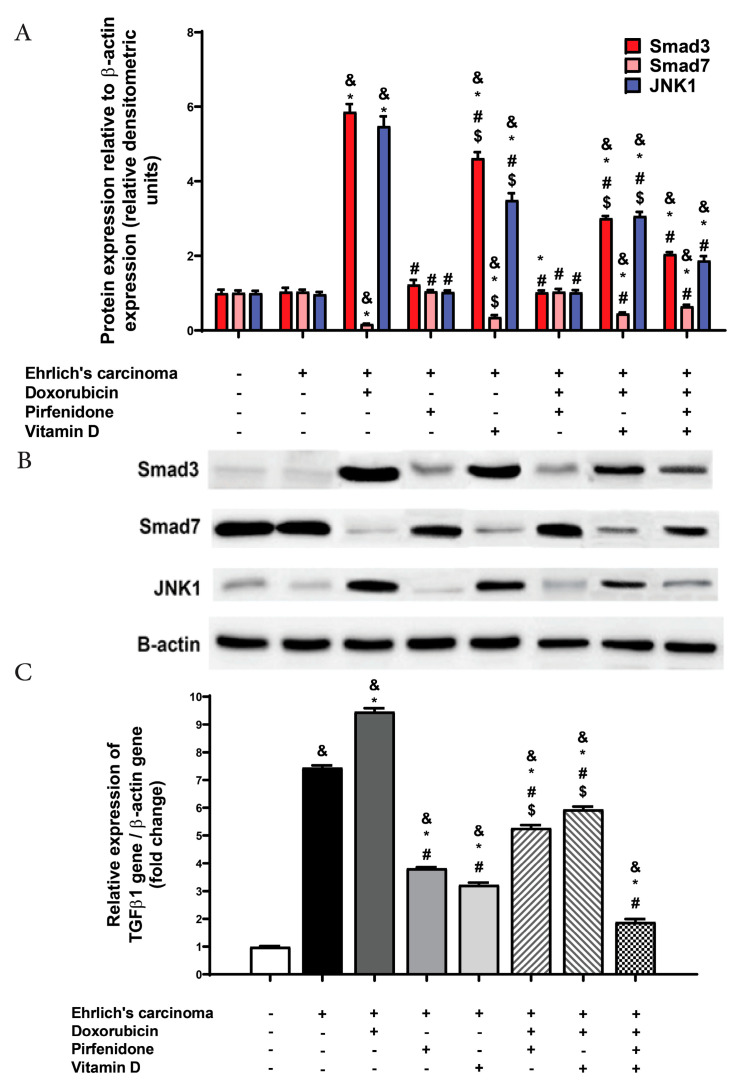
Effect of individual treatment with doxorubicin (15 mg/kg; i.p.), pirfenidone (500 mg/kg; p.o.) or vitamin D (0.5 µg/kg; i.p.) and their combinations on cardiac smad3, smad7 and JNK1 expressions in EAC bearing mice. (**A**) A graph showing quantitative analysis of the relative expression of different proteins after treatment. (**B**) Western blot analysis showing protein expression of cardiac smad3, smad7, and JNK1 and β-actin in different experimental groups. (**C**) RT-PCR measurement of TGF-β1 in cardiac tissue. ^&^ *p* < 0.05 with respect to normal, * *p* < 0.05 with respect to control EAC, ^#^ *p* < 0.05 with respect to doxorubicin. ^$^ *p* < 0.05 with respect to the co-therapy of doxorubicin, pirfenidone, and vitamin D (*n* = 5).

TGF-β1 is a profibrogenic cytokine acting for the recruitment of inflammatory cells and fibroblasts in the injured area. Smad3 is a marker of TGF-β1/Smad signaling pathways. In another way, smad7 is an inhibitor of the TGF-β1/Smad signaling pathways. Finally, JNK1 is considered as a pro-inflammatory and pro-fibrotic signaling pathway [16]. The obtained data revealed no significant change in the expression of smad3, smad7, and JNK1 between normal and control EAC groups. According to TGF-β1, it was found that the control EAC group and all treated groups showed a significant (*p* < 0.05) upregulation in their level in heart tissue compared with the normal group. Doxorubicin administration augmented TGF-β1, smad3, and JNK1 expressions significantly (*p* < 0.05) compared to both control EAC and normal groups. On the other hand, there was a significant (*p* < 0.05) downregulation in TGF-β1 expression following treatment with pirfenidone or vitamin D, either individually or in combination with doxorubicin compared to doxorubicin and control EAC groups. Furthermore, the co-therapy (doxorubicin, pirfenidone, and vitamin D) showed a remarkable (*p* < 0.05) decline in TGF-β1 expression compared to doxorubicin treated group, control EAC, and combination forms of doxorubicin with vitamin D or pirfenidone (Figure 3).

According to smad7, treatment with doxorubicin led to a significant (*p* < 0.05) decline in expression compared to control EAC and normal groups. On the other hand, all treated groups (except the combination form of pirfenidone and doxorubicin) showed augmentation in smad7 expression compared to doxorubicin. Individual treatment with either pirfenidone or vitamin D normalized smad7 expression. The co-therapy of (doxorubicin, pirfenidone, and vitamin D), showed a significant (*p* < 0.05) rise in smad7 expression compared to the combination form of doxorubicin and pirfenidone (Figure 3).

### 2.6. Effect on the Expression of α-SMA in Heart Tissue

**Figure 4:**
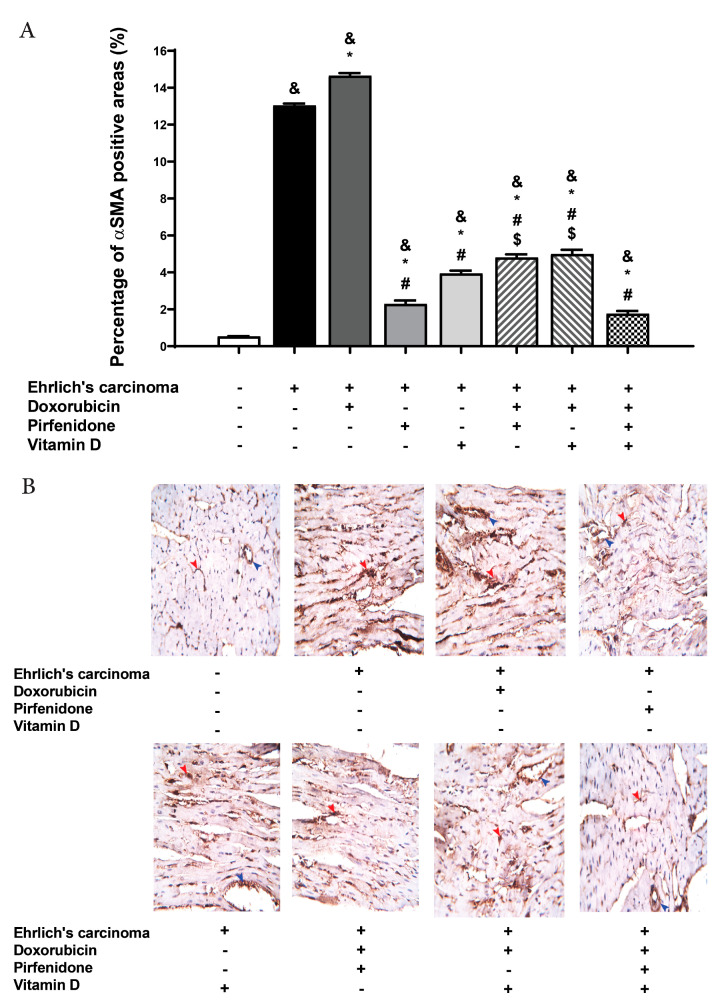
Effect of individual treatment with doxorubicin (15 mg/kg; i.p.), pirfenidone (500 mg/kg; p.o.) or vitamin D (0.5 µg/kg; i.p.) and their combinations on the cardiac expression of α-SMA in EAC bearing mice on day 21. (**A**) A graph showing the density of immunostaining for α-SMA in experimental groups. (**B**) Photomicrographs showing cardiac expression of α-SMA. ^&^ *p* < 0.05 with respect to normal, * *p* < 0.05 with respect to control EAC, ^#^ *p* < 0.05 with respect to doxorubicin. ^$^ *p* < 0.05 with respect to the co-therapy of doxorubicin, pirfenidone, and vitamin D (*n* = 5).

The expression of α-SMA stained cells was evaluated in tissue as a marker of fibrosis. The control EAC group showed a significant (*p* < 0.05) increase of α-SMA compared to the normal group. Doxorubicin treated group showed a remarkable (*p* < 0.05) increase in α-SMA compared to control EAC and normal groups. In groups treated with pirfenidone, vitamin D, either individually or in combination with doxorubicin, showed a significant (*p* < 0.05) decrease in α-SMA compared to the doxorubicin group and control EAC but this decrease failed to reach a normal level. In addition, the co-therapy of doxorubicin, pirfenidone, and vitamin D showed a significant (*p* < 0.05) decline in α-SMA compared to the doxorubicin group, control EAC, and combination forms of doxorubicin with pirfenidone or vitamin D (Figure 4).

### 2.7. Masson Trichrome (MT) Stain for Heart Tissue

**Figure 5:**
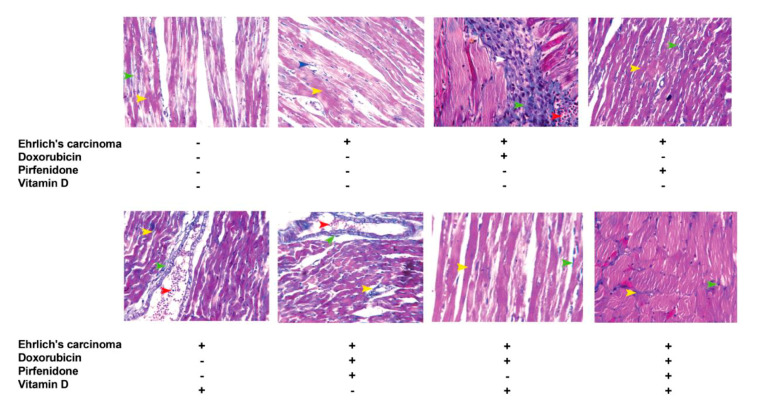
Representative photomicrographs for sections from the heart showing the effect of individual treatment with doxorubicin (15 mg/kg; i.p.), pirfenidone (500 mg/kg; p.o.) or vitamin D (0.5 µg/kg; i.p.) and their combinations on cardiac tissues fibrosis assessed by Masson trichrome staining in EAC bearing mice on day 21.

Control EAC group (A) showed red or dark red normal branched muscle fibers and normal few blue myocardial interstitial collagen fibers between the muscle bundles. Interestingly, the doxorubicin group (B) showed red, dark red (pink) and remarkably increased blue myocardial interstitial collagen fibers between the muscle bundles as well as numerous mononuclear pro-inflammatory cells infiltrated between muscles fibers. Also, it showed strong myocardial interstitial fibrosis (green arrowhead) as well as numerous mononuclear pro-inflammatory cells infiltrated between muscle fibers (white arrowhead) and a few extravagated RBCs. Pirfenidone (C) and vitamin D (D) treated groups showed red or dark red normal branched muscle fibers and few normal blue myocardial interstitial collagen fibers between the muscle bundles. Otherwise, the combination forms of doxorubicin with pirfenidone (E) or vitamin D (F) treated groups showed weak to moderate myocardial interstitial fibrosis with some mononuclear pro-inflammatory cell infiltration. Moreover, the co-therapy of doxorubicin, pirfenidone, and vitamin D treated group (G) showed red or dark red normal branched muscle fibers and very few blue myocardial interstitial collagen fibers between the muscle bundles (similar to the normal group) with slight mononuclear cells infiltration. Also, this combination showed very weak to normal myocardial interstitial and perivascular fibrosis with slight vessel congestion (Figure 5).

## 3. Discussion

Although the benefits of doxorubicin in reducing the mortality of patients with breast cancer have been demonstrated, doxorubicin induces late-onset cardiomyopathy and subsequent heart failure. Toxic cardiac effects of doxorubicin are considered major clinical challenges, especially as it is widely used in the oncology field. [17,18]. Previous studies demonstrated that acute cardiotoxicity can be induced by doxorubicin with a single dose of 15 mg/kg [19]. In the current study, treatment with pirfenidone or vitamin D decreased tumor weight and volume compared to the control EAC group. In agreement with this result, pirfenidone was explained to disrupt tumor–stromal interactions by inhibiting the effect of platelet-derived growth factor (PDGF-A), hepatocyte growth factor (HGF), collagen type I, and fibronectin which all play an important role in tumor–stromal interactions [20]. Furthermore, pirfenidone improved drug delivery and the efficacy of anti-cancer therapy by decompressing tumor blood vessels and increasing vessel perfusion through reducing collagen levels [21]. In addition, pirfenidone was found to significantly suppress the production of periostin which is involved in the recruitment and binding to osteoclast precursors [22]. Moreover, periostin is predominantly found in collagen-rich fibrous connective tissues in multiple organs and promotes tumor growth and metastasis [23]. Clinical trials showed the role of vitamin D and its metabolites in the inhibition of proliferation and differentiation of various carcinogenic cells [24]. In addition, vitamin D was shown to be able to work as an anti-tumor agent via several mechanisms including the suppression of tumor angiogenesis, invasion, and metastasis [25]. Interestingly, other studies have shown that vitamin D is able to target the estrogen signaling pathway in malignant breast epithelial cells via downregulation of the culprit protein, ERα [26]. Furthermore, the antitumor effects of vitamin D have been investigated in mice transplanted with human breast cancer cells [27].

In the present study, an elevation in cardiac troponin T and serum creatine kinase was detected in the control EAC group. Cardiac troponin is essential for prognosis in myocardial infarction and other cardiac diseases [28]. It has been revealed that EAC causes the suppression of the mammalian target of rapamycin (mTOR), a main signaling protein responsible for cell growth maintenance. This suppression is the major cause of cardiomyopathy associated with this type of cancer [29].

In experimental studies, doxorubicin increased the levels of cardiac biomarker enzymes such as cardiac troponin and creatine kinase [30]. Furthermore, treatment with doxorubicin increases heart and left ventricle weights due to the pressure overload and left ventricular (LV) hypertrophy, which causes LV systolic and diastolic dysfunction [31]. It is widely accepted that the cardiac toxicity of doxorubicin is mediated by reactive oxygen species [30]. These findings came in line with the results obtained in the current study that demonstrated the cardiotoxicity of doxorubicin.

Treatment with pirfenidone or vitamin D demonstrated significant improvement regarding the doxorubicin-induced cardiotoxicity represented by marked reduction in (CK, cTnT) levels and heart weight. This may be attributed to the suppression of cardiac fibroblast activation and collagen synthesis by pirfenidone treatment [32]. Also, clinical studies exposed that vitamin D produced a significant regression of the LV mass index and myocardial hypertrophy [33].

The inflammation process, which is closely related to fibrosis, contributes to the release of a large range of inflammatory mediators that can contribute to either the induction of fibrosis (profibrotic) or the suppression of fibrosis (antifibrotic). Chronic inflammation, notably in the disease state, is characterized by a prolonged inflammatory response, destruction of tissues, and release of many inflammatory cytokines such as TNF-α [34,35].

In the current study, the control EAC group showed an increase in the inflammatory mediators like NF-κB and MCP-1 while, doxorubicin-induced inflammation through upregulation of NF-κB and MCP-1 expressions. As the tumor progressed, blood leukocytes increased, and erythrocytes decreased leading to a sustained increase in inducible nitric oxide synthase (iNOS), cyclo-oxygenase (COX1), and IL-10 [36]. Doxorubicin activates NF-κB translocation to the nucleus via activating the degradation of its inhibitory protein IkB-α [37]. This stimulation of the NF-κB pathway is followed by the reproduction of inflammatory mediators, including TNF-α and MCP-1 [38]. Consequently, MCP-1 induces the expression and release of other inflammatory cytokines, infiltration of macrophages, and other inflammatory cells into cardiac tissues [39].

In the present study, pirfenidone and vitamin D downregulated MCP-1 and NF-κB. It has been reported that pirfenidone is able to block NF-κB and to inhibit the induction of iNOS gene expression via its intervention with NF-κB DNA binding [40]. Furthermore, pirfenidone inhibited MCP-1 by blocking the induction CCL2/chemokine signaling pathway [41,42]. Vitamin D possessed anti-inflammatory actions through the inhibition of NF-κB which is a main player transcription factor that regulates gene expression of several inflammatory molecules [43].

The final and typical pathological endpoint of many chronic inflammatory diseases is fibrosis. TGF-β1 is the master regulator molecule of fibrosis. TGF-β1 is a multifunctional cytokine that manages collagen formation and the deposition of collagen proteins in cardiac fibroblasts. Moreover, TGF-β1 is the principal controller of extracellular matrix protein deposition [44]. It has been suggested that TGF-β1 causes left ventricular systolic and diastolic dysfunction which results from left ventricular hypertrophy induced by pressure overload [45].

Consistent with these reports, the EAC control group showed upregulation of TGF-β1 gene expression, this can be ascribed to the secretion of TGFβ-like activity to extracellular medium in a partially-activated form [46].

It has been proposed that fibrosis is involved in cardiac stiffness and dysfunction in doxorubicin-induced cardiotoxicity [47]. Fibroblasts overproduce collage which then takes over and replaces the necrotic or apoptotic muscle cells [48]. In the present study, it was observed that doxorubicin-induced fibrosis through increasing collagen deposition, high expression of TGF-β1, α-SMA, smad3, and JNK1. Several studies were in agreement with this [6,7]. Not only TGF-β1 is a central mediator in fibrosis, but it acts also as apromoter for organ fibrosis via facilitation of myofibroblasts differentiation and induction ofz epithelial–mesenchymal transition [49]. Additionally, TGF-β1 increased α-SMA expression and induced endothelial mesenchymal transition (EMT), promoting a shift to a higher ratio of myofibroblasts [50].

Smad signaling has been recognized as the major pathway of TGF-β1 activity in progressive fibrosis. The protein “smad3” is considered the master mediator responsible for the biological outcomes of TGF-β1 [18]. On the other hand, smad7 operates as the functional antagonist of TGF-β1 signaling by interfering with R-Smad anchoring to their receptors [51,52].

JNK1 is considered another pro-inflammatory and pro-fibrotic mediator signaling protein due to its role in activating the enzymes that produce the latent form of TGF-β1. In addition, JNK1 phosphorylates smad3, targeting the final transcription of pro-fibrotic molecules [53]. There is a relationship between activated monophosphate kinase (AMPK) and the JNK1 pathway [54]. The oxidative stress produced by doxorubicin possesses a vital role in fibrosis development via activation of TGF-β1 and consequently, the mitogen-activated protein kinase (MAPK) pathway and the release of JNK1 [55]. Furthermore, doxorubicin enhanced the AMPK signaling due to the bioenergetics failure, genotoxic stress, oxidative stress, increase in energetic stress, and hypertrophy [56].

In the current study, it was noticed that doxorubicin-induced fibrosis was inhibited by pirfenidone and vitamin D via the attenuation of TGF-β1, α-SMA, smad3, and JNK1 expressions. On the other hand, the upregulation of smad7 has been recorded. Pirfenidone treatment ameliorated fibrosis through suppression of TGF-β1 gene transcription and decreasing TGF-band collagen type1 mRNA expression [57]. Moreover, pirfenidone targeted TGF-β/Smad3 signaling which represented a specific and effective therapy for fibrosis [58]. Besides, pirfenidone treatment inhibited the activation of MAPK and the induction of EMT [59].

Vitamin D was shown to possess an inhibitory effect on collagen and fibronectin synthesis and also on TGF-β1-induced EMT and extracellular matrix accumulation [60]. Vitamin D was also shown to inhibit the TGF-β/Smad pathway [61]. Another study showed that the beneficial effects of vitamin D on TGF-β1 signaling is related to its ability to reduce the expression of extracellular signal-regulated kinase 1/2 and JNK1 [62].

In summary, the addition of pirfenidone and vitamin D to doxorubicin markedly improved doxorubicin-induced cardiac fibrosis compared to sole treatment with either pirfenidone or vitamin D.

## 4. Materials and Methods

### 4.1. Animals

Female albino mice with an initial weight ranging from 20 to 25 g were used. The Modern Veterinary Office for Laboratory Animals (Cairo, Egypt) was the provider for all mice. Mice had been accommodated under a temperature of (25 ± 1 °C) on a 12-h light:12-h dark cycle. Food and water were allowed ad libitum during the whole investigation period. The research protocol has been approved (License number 201805PHDA1) by the research ethics committee at the Faculty of Pharmacy, Suez Canal University (Ismailia, Egypt) in agreement with the Guidelines of the Canadian Council on Animal Care.

### 4.2. Drugs

Pirfenidone was obtained from (Sigma Pharmaceuticals Company, Quesna, Egypt) and administrated orally and daily at a dose level of 500 mg/kg body weight [21]. Vitamin D was obtained from Medical Union Pharmaceuticals (Abu sultan, Ismailia, Egypt) and administrated daily via the intraperitoneal route at a dose of 0.5 μg/kg body weight [63].

### 4.3. Ehrlich’s Ascites Carcinoma Cell Line and Induction of In Vivo Solid Tumors

From the Department of Tumor Biology, National Cancer Center, Cairo University, the Ehrlich’s ascites carcinoma (EAC) cell line was obtained. EAC is spontaneous murine breast cancer that acted as the tumor from which an ascites variant was obtained. An ascitic fluid abundant with tumor cells was obtained via intraperitoneal inoculation. Maintenance of the tumor cell line was accomplished in our laboratory by serial intraperitoneal passage into female albino mice at seven- to ten-day intervals. The EAC cells were obtained under strict aseptic conditions. Contamination and viability of EAC cells were evaluated utilizing the Trypan blue dye exclusion technique [64]. EAC cells were suspended in a concentration of 2.5 × 10^6^ EAC cells per 0.1 mL of normal saline. A hemocytometer was used to count the cells under the microscope. Each mouse was inoculated intradermally with 100 μL EAC suspension (2.5 × 10^6^ cells) at the two bilateral sides on the lower ventral region.

### 4.4. Induction of Fibrosis

Fibrosis was induced by the administration of a single dose of doxorubicin intraperitoneal (15 mg/kg/bodyweight) [65].

### 4.5. Experimental Design

Eight groups were created with 10 mice each. Groups were treated as follows: the first group received saline and served as normal. Groups 2 to 8 all received EAC cells. The second group acted as a control EAC and received only saline. The third group received a single dose of doxorubicin. The fourth group received pirfenidone. The fifth group received vitamin D. The sixth and seventh groups were treated with (doxorubicin and pirfenidone) and (doxorubicin and vitamin D), respectively. The eighth group received a combination of doxorubicin, pirfenidone, and vitamin D. All treatments had begun one week after tumor inoculation, which was considered as day zero and continued daily for 14 days.

### 4.6. Collection of Blood and Tissue Samples

At the end of the experiment, mice were given thiopental sodium (50 mg/kg) and then sacrificed. Blood was collected via cardiac puncture in a dry Eppendorf tube. Blood samples were allowed to set for 30 min. Blood samples were then centrifuged at 2000× *g* for 15 min. Serum was separated, collected in separate Eppendorf tubes, and then stored at −20 °C until use in biochemical analyses. Hearts were isolated and collected in ice-cold phosphate-buffered saline (pH = 7.4). Hearts were cut longitudinally into two parts; the left ventricle myocardium was fixed in paraformaldehyde solution and used in histopathological investigations. The remaining heart tissues were frozen at −80 °C (for Western blotting and RT-PCR analyses).

### 4.7. Determination of Serum Troponin T

Serum troponin level was determined using an enzyme-linked immunosorbent assay (ELISA) using mouse cardiac troponin (ELISA kit) that was purchased from R&D systems^®^ (Minneapolis, MN, USA) following the manufacturer’s protocol. In brief, to a 96-well microplate pre-coated with a monoclonal antibody specific for mouse cTnT, 50 μL of assay diluent and 50 μL of control, or sample was added per well. Mouse cTnT standards were used to construct the standard curve. After incubation for 2 h at room temperature, the wells were washed and 100 μL of mouse cTnT conjugate was added to each well. Incubation continued for 2 h and the plate was washed, then 100 μL of substrate solution was added to each well, incubated for 30 min at room temperature, and protected from light. Finally, 100 μL of stop solution was added to each well and the optical density of each well was determined within 30 min, using a microplate reader set to 450 nm.

### 4.8. Determination of Serum Creatine Kinase (CK)

Serum creatine kinase levels were determined using a colorimetric CK kit from Biodiagnostic (Giza, Egypt) following the manufacturer’s protocol.

### 4.9. Determination of Serum Creatine Kinase (CK-MB)

Serum creatine kinase levels were determined using an ELISA following the manufacturer’s protocol (MBS008782, My Biosource, San Diego, CA, USA).

### 4.10. Determination of TGF-β1 and MCP-1

TGF-β1 and MCP-1 gene expression was evaluated using reverse transcription-polymerase chain reaction (RT-PCR). In brief, pure RNA was extracted using a total RNA Purification Kit according to the manufacturer’s protocol (Thermo Scientific, Fermentas, #K0731). A high capacity cDNA reverse transcription kit was utilized to convert the total RNA (0.5 to 2 µg) to cDNA. The cDNA samples were then stored at −20 °C. The isolated cDNA was amplified using 2X Maxima SYBR Green/ROX qPCR Master Mix following the manufacturer’s protocol (Thermo Scientific, Waltham, MA, USA). The qRT-PCR assay with the following gene-specific primer sets was optimized with the annealing temperature (MCP-1; forward primer: GCAGCAGGTGTCCCAAAGAA, reverse primer: ATTTACGGGTCAACTTCACATTCAA, TGF-β1; forward primer: GCAACATGTGGAACTCTACCAGA, reverse primer: GACG TCAAAAGACAGCCACTCA, β-actin; forward primer: ACTATTGGCAACGAGCGGTT, reverse primer: CAGGATTCCATACCCAAGAAGGA). Real-time PCR amplification and analysis were performed to measure the expression of mRNAs of target genes in the tissue relative to β-actin mRNA expression as an internal reference.

### 4.11. Determination of smad3, samd7, and JNK1

Cardiac tissued which had been stored at −80 °C were lysed and then allowed to remain on ice for 30 min. Lysates were then centrifuged for 30 min at 15,000 rpm at 4 °C. The traditional western protocol was followed. The polyvinylidene fluoride membranes were then incubated with specific primary antibodies including anti-smad3, smad7, and JNK1 (Santa Cruz, CA, USA). On the next day, the β-actin monoclonal antibody (Santa Cruz, CA, USA) was added and incubated for 1 h. All membranes were then incubated with the appropriate secondary antibody (horseradish peroxidase-conjugated goat anti-rabbit IgG). Image J software was utilized to analyze the protein of interest band densities and normalized to that of the β-actin protein band.

### 4.12. Immunohistochemistry

Cardiac sections at 4-μm thick were prepared in order to perform immunostaining. Sections were first deparaffinized followed by the antigen retrieval which was processed by heating section slides in 0.01 M citrate buffer solution (pH = 6.0) for 15 min. Primary antibodies against NF-κB p105/p50 (bs-1194R) (Bioss Antibodies, Woburn, MA, USA) and against α-SMA (Clone 1A4, Sigma-Aldrich, St. Louis, MO, USA) were added to the slide sections for overnight incubation. Positive staining was detected by mixing 40 µL of 3,3′-diaminobenzidine with chromogen in 2 mL of 3,3′-diaminobenzidine plus substrate. The mixture was then applied to tissue sections and incubated for 5 to 15 min then rinsed in PBS three times for 2 min each. Counterstaining was attained by using Mayer’s hematoxylin. Slides were then rinsed in running tap water for 10 min, dehydrated through 95% ethanol for 1 min, 100% ethanol two times for 3 min, and cleared in xylene two times for 5 min. The positively stained area was brown in color and was observed against the negatively stained region. Finally, color intensity was estimated using an image analyzer (Image J program).

### 4.13. Histopathological Examination of Heart Tissue

Other cardiac sections (3-μm thick) were stained with Masson trichrome (MT). Heart sections were fixed in formalin–alcohol for 48 h, dehydrated in serial alcohol concentrations, processed in xylene, embedded in paraffin, and finally dried. Each section was first generally examined at low power (×10 magnification) whereas scoring and the final read-out were performed at high power (×40 magnification).

### 4.14. Statistical Analysis

All data are represented as mean ± SEM. Statistical significance was calculated by one-way analysis of variance (ANOVA) followed by Bonferroni’s post-hoc analysis. A *p*-value < 0.05 was set as the level of significance.

## 5. Conclusions

Current data demonstrated a new mechanism by which pirfenidone and vitamin D provoked an anti-fibrotic effect in mice through the inhibition of JNK1 and MCP-1 pathways. This lends further evidence to the anti-fibrotic potential of pirfenidone and vitamin D representing an attractive approach to combat cardiotoxicity of doxorubicin by interfering with several pathways involved in cardiac fibrosis. Our study is paving the way for the use of the combined therapy of doxorubicin, pirfenidone, and vitamin D in guidelines that include doxorubicin as a treatment for different kinds of tumors.

## Figures and Tables

**Table 1:** Effect of doxorubicin (15 mg/kg; i.p.) and/or pirfenidone (500 mg/kg; p.o.), vitamin D (0.5 µg/kg: i.p.) on heart, left ventricle, tumor weight, and tumor volume for all groups two weeks starting from day 8 after EAC implantation. * *p* < 0.05 with respect to control EAC, ^&^ *p* < 0.05 with respect to normal, ^#^ *p* < 0.05 with respect to doxorubicin. ^$^ *p* < 0.05 with respect to the co-therapy of doxorubicin, pirfenidone, and vitamin D (*n* = 5).

Treatment Group	Heart Weight (gm)	Left Ventricle Weight (gm)	Tumor Weight (gm)	Tumor Volume (cm^3^)
Normal	0.09 ± 0.004			
Ehrlich’s carcinoma	0.09 ± 0.005	0.04 ± 0.004	0.26 ± 0.01	0.45 ± 0.04
Ehrlich’s carcinoma + Doxorubicin	0.13 ± 0.004 *^,&^	0.07 ± 0.004 *	0.17 ± 0.01 *	0.25 ± 0.01 *
Ehrlich’s carcinoma + Pirfenidone	0.09 ± 0.002 ^#^	0.04 ± 0.002 ^#^	0.21 ± 0.02 ^$^	0.27 ± 0.02 *^,$^
Ehrlich’s carcinoma +Vitamin D	0.09 ± 0.003 ^#^	0.04 ± 0.002 ^#^	0.18 ± 0.01 *^,$^	0.27 ± 0.02 *^,$^
Ehrlich’s carcinoma + Doxorubicin + Pirfenidone	0.1 ± 0.008 ^#^	0.06 ± 0.002 ^#^	0.15 ± 0.01 *	0.19 ± 0.01 *
Ehrlich’s carcinoma + Doxorubicin + Vitamin D	0.11 ± 0.005	0.04 ± 0.004 ^#^	0.14 ± 0.01 *	0.18 ± 0.01 *
Ehrlich’s carcinoma + Doxorubicin + Pirfenidone + Vitamin D	0.09 ± 0.004 ^#^	0.04 ± 0.004 ^#^	0.12 ± 0.01 *	0.15 ± 0.01 *
